# Mechanistic insights and optimization strategies for perovskite single-crystal thin film growth

**DOI:** 10.1039/d4sc08145e

**Published:** 2025-02-24

**Authors:** Jingyi Sun, Runda Li, Yang Gui, Xinyi Shao, Jingjing Xue, Rui Wang

**Affiliations:** a State Key Laboratory of Silicon and Advanced Semiconductor Materials, School of Materials Science and Engineering, Zhejiang University Hangzhou 310027 China jjxue@zju.edu.cn; b School of Engineering, Westlake University and Institute of Advanced Technology, Westlake Institute for Advanced Study Hangzhou 310024 China wangrui@westlake.edu.cn; c Shangyu Institute of Semiconductor Materials Shaoxing China; d Division of Solar Energy Conversion and Catalysis at Westlake University, Zhejiang Baima Lake Laboratory Co. Ltd Hangzhou China; e Zhejiang Provincial Key Laboratory of Intelligent Low-Carbon Biosynthesis, Westlake University Hangzhou China

## Abstract

Perovskite materials, with their tunable band gaps, high optical absorption, and excellent carrier mobility, are key candidates for lasers, LEDs, photodetectors, and solar cells. Polycrystalline thin films dominate current applications but suffer from efficiency and stability losses largely due to grain boundaries. Perovskite single-crystal thin films (SCTFs) offer optimized carrier diffusion and reduced recombination losses, though challenges in achieving high-quality SCTFs remain. Fabrication techniques and device applications of SCTFs have been widely explored, yet the crystallization mechanisms that critically influence film quality and device performance offer significant opportunities for further investigation. This review aims to provide a comprehensive analysis of SCTF nucleation, growth dynamics, and structural optimization, highlighting the role of external factors like substrate properties and solution chemistry. By advancing the understanding of these mechanisms, we hope to guide efficient SCTF fabrication and inspire innovations in high-performance, stable perovskite-based optoelectronics.

## Abstract 2

Perovskite materials, with their tunable band gaps, high optical absorption, and excellent carrier mobility, are key candidates for lasers, LEDs, photodetectors, and solar cells.

## Graphical abstract

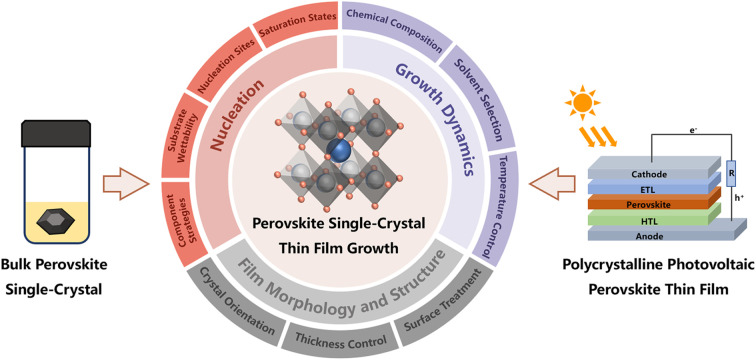

## 1. Introduction

Perovskite materials have garnered immense attention in recent years due to their unique structure and outstanding optoelectronic properties.^1^ Perovskite materials exhibit tunable band gaps, high optical absorption coefficients, and large carrier mobility. These features allow for extensive tunability, establishing perovskite material as a research hotspot in lasers,^2,3^ light-emitting diodes (LEDs),^4,5^ photodetectors,^6^ and solar cells.^7^ While polycrystalline perovskite thin films (PCTFs) have achieved remarkable success in practical applications, their performance is limited by the presence of numerous grain boundaries. These boundaries act as defect sites, leading to non-radiative recombination and charge trapping, ultimately resulting in energy loss and reduced device efficiency and stability.^8–10^ For instance, grain boundaries have been shown to decrease the carrier lifetime to less than 100 ns in PCTFs, compared to several microseconds in single-crystal counterparts. Moreover, charge trapping at grain boundaries can lower the open-circuit voltage of solar cells by over 200 mV, significantly impacting device performance.^11,12^

In contrast, single-crystal perovskites, with their grain-boundary-free structure and lower defect density, offer higher theoretical efficiency potential.^1,13^ Single crystal perovskites exhibit carrier mobilities exceeding 1000 cm^2^ V^−1^ s^−1^, an order of magnitude higher than the typical 10–20 cm^2^ V^−1^ s^−1^ observed in PCTFs. This improved mobility facilitates more efficient charge transport, reducing non-radiative recombination and enhancing photogenerated current. For example, recent studies on perovskite single-crystal thin films demonstrate external quantum efficiencies exceeding 90%, a value difficult to achieve with PCTFs due to charge losses at grain boundaries.^14,15^ Additionally, Perovskite SCTFs show significantly better long-term stability, maintaining over 90% of their initial performance after 1000 hours of operation, compared to polycrystalline films, which often degrade under similar conditions.^16,17^ It is notable that most reported single-crystal perovskites are fabricated as bulk crystals several millimeters thick, which significantly exceed the carrier diffusion length, leading to pronounced recombination losses that hinder their performance in optoelectronic applications.^18^ Additionally, bulk crystals pose challenges in thickness control and fabrication complexity, making it difficult to achieve large-area uniform coverage, further restricting their utility in practical devices. Perovskite single-crystal thin films combine the advantages of single-crystal and polycrystalline thin films, retaining the grain-boundary-free structure of single crystals while offering the processability and scalability typically associated with polycrystalline films, thus overcoming the challenges posed by the excessive thickness of bulk crystals.

Several methods have been developed to fabricate perovskite SCTFs, including cavitation-triggered asymmetric crystallization,^19^ slicing of bulk single crystals^20^ and space-confined inverse temperature crystallization.^21–24^ Recently, *in situ* growth of perovskite SCTFs on hole transport layers (HTLs) has emerged as a promising strategy. This approach facilitates tighter interfacial contact, reduces charge transport losses, and improves charge separation efficiency.^25^ However, since perovskite crystals exhibit isotropic growth in solution, limiting thickness inherently restricts lateral growth.^4,26^ Consequently, achieving high-quality SCTFs with a high area-to-thickness ratio (ATT) on HTLs remains a critical challenge that requires further exploration.

Nucleation dynamics, crystal growth behaviors, and various external factors—including substrate surface properties, solution chemistry, and thermal conditions—play critical roles in determining the quality and morphology of SCTFs. Achieving high-quality SCTFs necessitates an in-depth understanding of these growth mechanisms. However, while many reviews on perovskite SCTFs have highlighted advancements in fabrication methods and device applications, detailed discussions on the fundamental growth mechanisms deserve further exploration. Since both nucleation and subsequent crystal growth directly influence the structural and morphological characteristics of SCTFs, these processes critically affect the efficiency and stability of optoelectronic devices. Understanding these growth mechanisms not only helps identify and address limiting factors but also offers theoretical guidance for optimizing parameters and methodologies, enabling higher-quality SCTFs and addressing key challenges in perovskite device performance and long-term stability.

Herein, in this review, we discuss the crystallization mechanisms of high-quality perovskite SCTFs, summarizing the latest progress in crystal nucleation, growth dynamics, and structural optimization, including thickness and orientation control. We highlight the critical role of factors such as saturation control, nucleation site modulation, and chemical composition optimization in determining film quality. By providing theoretical support for the efficient fabrication of SCTFs, we hope to provide new insights for developing high-performance, stable perovskite-based optoelectronic devices.

## 2. Nucleation

The nucleation stage preceding the growth of perovskite SCTFs determines the initial conditions for crystal formation. The goal of this stage is to achieve precise control over the nucleation process, ensuring that crystals exhibit high quality from their initial formation, which ultimately impacts the morphology and optoelectronic performance of the thin films. The unique ionic nature and chemical properties of the solution of perovskites result in significant differences in their nucleation process, both kinetically and thermodynamically.^27,28^ These properties set perovskites apart from other materials and contribute to the rapid crystallization process under mild conditions. The following equations help describe key aspects of the nucleation process in perovskites.

Supersaturation (Δ*C*) plays a critical role in nucleation, defined as the difference between the actual concentration of solute and its equilibrium concentration:1
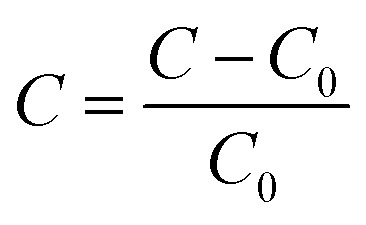
where *C* and *C*_0_ are the actual and equilibrium solute concentrations, respectively. As temperature increases, perovskites experience inverse solubility behavior, meaning that their solubility decreases with rising temperature. This leads to higher supersaturation, which facilitates nucleation. For instance, in inverse temperature crystallization, the temperature is gradually increased, enhancing the supersaturation and thus promoting more uniform nucleation, which improves film quality and uniformity.

The relationship between supersaturation and the nucleation energy barrier is expressed by:2
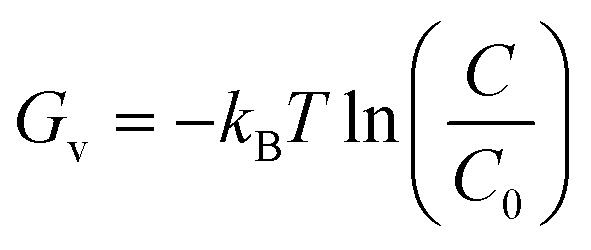
where *k*_B_ is the Boltzmann constant, *T* is the temperature, and Δ*G*_v_ represents the volume free energy change that determines the difficulty of nucleation. In perovskites, the surface energy (*γ*) and ion migration (*e.g.*, PbI_2_ complexes with organic solvents like DMF or DMSO) significantly influence Δ*G*_v_. These factors reduce surface tension at the nucleation interface, lowering the nucleation energy barrier and promoting faster nucleation at lower supersaturation compared to other materials. However, this also introduces the challenge of excessive nucleation, which leads to a higher density of grain boundaries, negatively impacting film quality.

The inverse solubility of perovskites complicates this process further: as temperature increases, solubility decreases, leading to higher supersaturation. This property enables temperature regulation for controlled nucleation. For instance, inverse temperature crystallization gradually increases supersaturation with rising temperature, leading to more uniform nucleation and improved film quality.^29^

The nucleation process also depends on the critical nucleus radius (*r*_c_), which determines whether a nucleus will grow or dissolve. The critical radius is given by:3
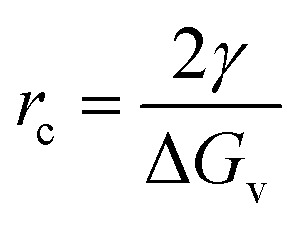
where *γ* is the surface energy, and Δ*G*_v_ is the volume free energy change. A smaller *r*_c_ favors nucleation, but it can also increase the number of small nuclei, which may lead to more grain boundaries. Therefore, controlling the nucleation density and ensuring uniform growth is crucial for high-quality films. In perovskites, lower interfacial energy and higher supersaturation reduce *r*_c_, promoting nucleation. However, smaller nuclei may increase grain boundaries, requiring a balance between nucleation density and uniform growth.

The nucleation rate, which determines how quickly stable nuclei form in a supersaturated solution, can be expressed in different ways, depending on the aspects being emphasized. First, we can describe the nucleation rate *J*_0_ in terms of the initial kinetics:

4 *J*_0_ = N* × *N*_1_ × ω* × *Γ*

Where N* represents the equilibrium concentration of critical nuclei, *N*_1_ denotes the monomer concentration in the solution, ω* is the frequency of monomers attaching to nuclei, and *Γ* is the Zeldovich factor. This equation emphasizes the dynamic factors influencing nucleation, particularly during the early stages, such as monomer concentration and attachment frequency. In the case of perovskites, surface energy and ion mobility—which are influenced by the chemical composition of the precursor solution—play a critical role in determining the number and stability of nucleation sites.^30–32^ To calculate the total energy barrier *G* for nucleation, we use the equation:5
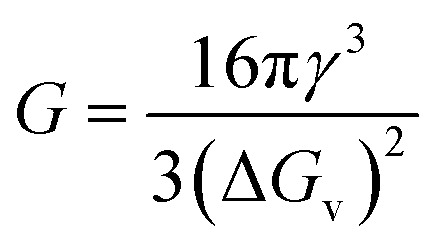
where *γ* is the surface energy, and Δ*G*_v_ is the volume free energy change. In perovskites, typical PbI_2_ and organic cations form complexes in solution, altering the local chemical environment to reduce the surface tension at the nucleation interface. This reduction in interfacial energy lowers the nucleation energy barrier, facilitating nucleation under milder conditions compared to other materials.^28,33,34^ This feature allows perovskites to nucleate rapidly at low supersaturation, facilitating efficient thin-film preparation. However, the reduced nucleation energy barrier may also lead to excessive nucleation, increasing grain boundaries and negatively affecting film optoelectronic properties.

The second expression for nucleation rate provides a more generalized description that incorporates both thermodynamic and kinetic factors, and can be described as follows:6
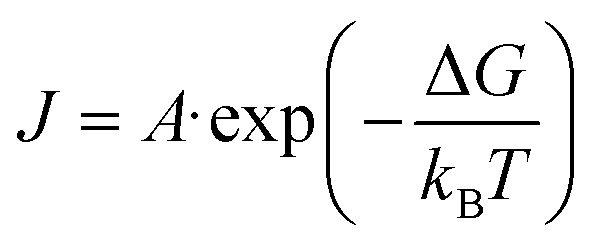
where *A* is a prefactor and Δ*G* represents the total Gibbs free energy barrier for nucleation, which accounts for both bulk thermodynamic driving forces (Δ*G*_v_) and surface energy contributions. This equation generalizes the nucleation rate by considering the total energy barrier, which includes both the bulk thermodynamic driving forces (Δ*G*_v_) and surface energy contributions. It offers a more comprehensive understanding of the nucleation process, particularly in relation to temperature and energy conditions. It is important to note that the nucleation rate is governed not only by the supersaturation (as seen in eqn (2)) but also by the overall energy barrier, which is a combination of thermodynamic and kinetic factors.

The distinctiveness of perovskites is also evident in the influence of ion migration on nucleation dynamics. For example, complexes of PbI_2_ with solvents like DMF or DMSO adjust *γ* and Δ*G*_v_, while regulating ion release rates to impact nucleation and growth.^34,35^ By optimizing the chemical composition, concentration, and temperature of the solution, the nucleation process can be finely tuned, enabling efficient growth of high-quality perovskite SCTFs.

These factors—supersaturation, nucleation energy barrier, nucleation rate, and critical nucleus radius—are central to understanding the nucleation process of perovskites. By optimizing these parameters, one can control the density of nucleation sites and ensure that crystals grow in a uniform, defect-free manner. The following sections will explore how these theoretical principles can be applied to component regulation, dopant strategies, and growth optimization methods, offering practical insights into the production of high-quality perovskite SCTFs.

### 2.1 Control of saturation states

The nucleation and growth of high-quality perovskite SCTFs are profoundly influenced by the states of saturation and supersaturation within the precursor solution. Precise control over these states not only governs nucleation rates but also directly determines the crystallinity and uniformity of the resulting thin films. Maintaining a quasi-saturated state during crystal growth helps regulate the nucleation speed, ensuring uniform growth while minimizing the risk of excessive nucleation that leads to uneven crystal development and increased structural defects, which can severely degrade the optoelectronic properties of SCTFs.

As is shown in eqn (1) and (2), key aspects of nucleation, such as supersaturation (Δ*C*) and the energy barriers associated with nucleation (Δ*G*_v_), were discussed. The control of saturation states, including the manipulation of supersaturation levels, is a critical strategy for optimizing the nucleation process in perovskite SCTFs, and allows for the creation of quasi-saturated solutions during crystal growth. By carefully regulating these parameters through temperature and solvent control, the nucleation rate can be fine-tuned to achieve uniform growth, minimizing defects and ensuring high-quality films.

Temperature-dependent solubility of perovskite components plays a critical role in this context. For instance, the solubility of methylammonium lead iodide (MAPbI_3_) at 60 °C is approximately 1.7 M, whereas FA_0.6_MA_0.4_PbI_3_ (FA^+^ is formamidinium cation) reaches a higher maximum solubility of 2.1 M around 50 °C.^36^ This discrepancy arises from the higher solubility of FAI compared to MAI in PbI_2_ solution, allowing solutions containing FAI to maintain a quasi-saturated state at higher concentrations.^37^ This feature facilitates nucleation at lower temperatures, enabling the preparation of low-defect SCTFs. By selecting appropriate organic cations, the solution's saturation state and solubility curve can be tuned to reduce the occurrence of oversaturation during cooling, which optimizes nucleation temperature and minimizes defect formation.

**Fig. 1:**
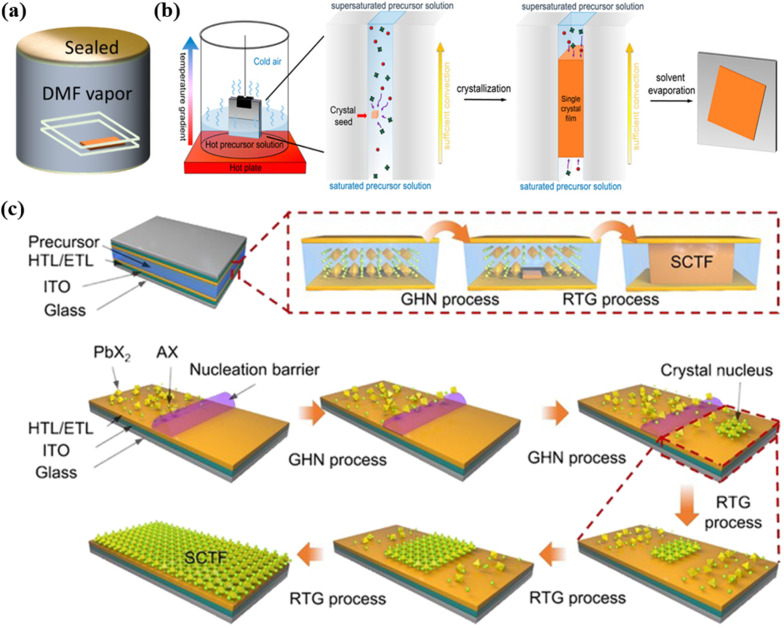
(a) Schematic diagram of saturated solvent vapor assisted method.^42^ (b) Bottom-up saturation techniques to induce crystallization.^38^ (c) Schematic of the nucleation and growth process of the FAPbBr_3_ SCTFs by room temperature gradient crystallization method.^46^

Controlled supersaturation can also be achieved through bottom-up saturation techniques, where the bottom layer of the solution remains saturated while the upper layer becomes supersaturated due to cooling, inducing crystallization, as depicted in Fig. 1b.^38^ This approach enables a gradual supersaturation process that regulates crystal growth rates and reduces nucleation density, yielding larger single-crystal regions. Dynamic adjustments to precursor vapor concentrations provide an even more refined method for controlling the relationship between vapor pressure and solution saturation, significantly enhancing film uniformity.^39,40^

Solvent properties such as polarity, evaporation rate, and solubility play pivotal roles in determining supersaturation dynamics, which in turn affect nucleation rates and crystal distribution. The proper solvent design and evaporation control can ensure a gradual transition to a quasi-saturated state, reducing localized nucleation rates. Commonly used DMF, for example, exhibits spatially uneven evaporation rates under specific conditions, with edge regions often reaching saturation earlier at elevated temperatures. Gradual heating strategies can mitigate this by suppressing rapid solvent evaporation, promoting uniform supersaturation across the substrate surface.^28,41^ Additionally, placing the growth environment in saturated DMF vapor can further balance evaporation rates and prevent premature edge nucleation, ensuring more consistent crystal growth across the thin film, as depicted in Fig. 1a.^42^

Moreover, the development of solvent systems with controllable evaporation rates or the inclusion of functional additives can optimize the nucleation process for perovskite SCTFs. Incorporating long-chain organic molecules or amphiphilic additives slows solvent evaporation while guiding crystals to grow along preferred directions.^27,39,40^ Recent studies on mixed solvent systems, such as DMF combined with DMSO, have demonstrated significant improvements in film crystallinity and defect suppression, underscoring the potential of advanced solvent engineering to enhance perovskite film quality.

### 2.2 Regulation of nucleation sites

The distribution of nucleation sites in perovskite crystals directly impacts the growth sequence and the number of grain boundaries, making precise control of nucleation sites critical for achieving uniform growth and large-size crystals in perovskite SCTFs. As previously discussed, the nucleation rate *J*_0_ and the nucleation energy barrier Δ*G*_v_ directly influence the formation of nucleation sites. By controlling the solubility and supersaturation of the precursor solution, we can regulate the nucleation rate and the formation of stable nucleation sites. The relationship between Δ*C* and Δ*G*_v_, expressed by eqn (1) and (2) highlights the thermodynamic and kinetic factors that drive nucleation. Localized heating can confine solubility to specific regions, ensuring that nucleation and growth primarily occur in these predesignated hot zones, enabling precise control over nucleation in specific areas.^21,41,43^

However, despite the benefits of localized heating, achieving uniform nucleation across larger areas with more complex structures remains challenging. To address this, it is necessary to manage not only the temperature but also the overall supersaturation and nucleation barriers across the entire substrate, as described in eqn (4) and (5). These equations show how the nucleation rate, influenced by both the monomer concentration and the Gibbs free energy barrier Δ*G*, governs the nucleation process and can be adjusted by fine-tuning the precursor solution's properties.

Thus, the control over nucleation site distribution is intricately linked to both the thermodynamic and kinetic parameters discussed in the previous formulas, and localized heating serves as an effective strategy to influence these parameters and optimize film growth.

In controlled microenvironments, further optimization of nucleation density and growth direction can be achieved by regulating temperature and saturation state dynamics. Inverse temperature crystallization, for instance, promotes the vertical alignment of nuclei relative to the substrate during the heating phase,^44,45^ potentially introducing grain boundaries with random orientations and degrading overall crystal quality. To address this issue, it is possible to form a single nucleus by lowering the heat source temperature, limiting nucleation density, and promoting the growth of large size single crystal films.^42^ The GHN-RTG (room temperature gradient crystallization) method depicted in Fig. 1c takes advantage of micro-supersaturation to optimize the nucleation process.^46^ Micro-supersaturation refers to a state where the precursor concentration slightly exceeds its equilibrium solubility, but not to the extent that spontaneous crystallization occurs. This condition creates fewer nucleation sites, ensuring that the nucleation is spatially confined to specific regions. In the GHN-RTG method, the temperature gradient induces localized supersaturation, which allows for the controlled formation of a small number of stable nuclei. Once these nuclei are formed, immediate cooling is applied to slow down the growth process and ensure that the growth proceeds uniformly from these few, stable nuclei.

By controlling nucleation density through micro-supersaturation, the GHN-RTG method minimizes the formation of random grain boundaries, ensuring that crystal growth occurs primarily from these fewer, stable nuclei. This method also reduces the supersaturation of the precursor solution, leading to the dissolution of smaller crystals through Oswald ripening, which refers to the phenomenon where smaller crystals dissolve and the dissolved material is redeposited onto larger crystals, leading to an increase in the size of the larger crystals and the elimination of smaller ones. This process ensures that only the larger, more stable crystals continue to grow, thereby improving the overall quality of the single crystal film.

**Fig. 2:**
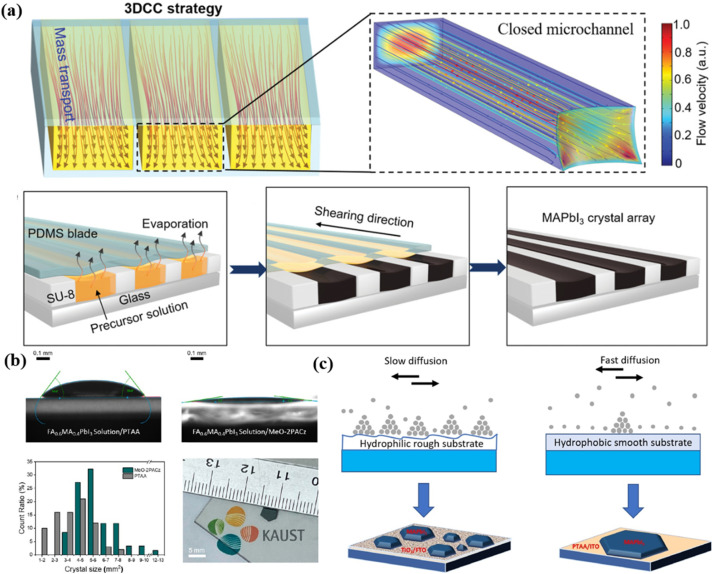
(a) Schematics of fluid flow and illustration of the growth process of perovskite crystals within the closed 3D microchannel.^47^ (b) Contact angle measurements and reproducibility counts of single crystals size on PTAA-coated ITO glass and MeO-2PACz-coated ITO glass.^55^ (c) Growth mechanism of MAPbI_3_ films on TiO_2_/FTO and PTAA/ITO substrates.^58^

Microchannel-based fluid control represents an innovative method for optimizing nucleation. The chaotic flow of solution over a substrate often leads to random molecular distribution and spontaneous nucleation. In contrast, the laminar flow within microchannels facilitates controlled nucleation distribution and growth direction. As depicted in Fig. 2a, the high solvent flow rate in microchannels enables rapid replenishment of solutes as they are consumed, significantly improving crystal growth efficiency and size.^47^ Despite its effectiveness in controlling nucleation and mass transfer, the design and manufacturing costs of microchannels may limit their scalability for industrial applications, particularly when considering the complexity and expense of producing large-area microchannel substrates.

On the other hand, space-confined growth methods, which involve restricting the growth space to control the size and distribution of crystals, offer a different set of advantages and challenges. While space confinement can lead to high-quality single crystals with minimized defects, scalability remains a critical issue. The ability to uniformly control the space and the interaction between precursors across larger areas is difficult to achieve, potentially limiting its use for large-scale production. Moreover, the integration of space-confined methods into continuous production systems, as well as the uniformity of the controlled environment, must be optimized to ensure high throughput and consistent quality.

Both techniques could benefit from advancements in substrate stability and the compatibility of inducers with precursor solutions. Microchannel methods, for instance, might see improvements through the integration of external field controls, such as light, electric, or magnetic fields, to enhance precision in nucleation. Similarly, space-confined growth could be enhanced by incorporating external fields to fine-tune nucleation rates, and combining microchannel designs with macro-scale nucleation controls could further stabilize the growth of large-area single-crystal films.^48,49^ Furthermore, ensuring the durability of substrate modifications or patterning processes is essential, especially considering the sensitivity of perovskite materials to heat and humidity. The development of more robust, adaptable substrates and scalable fabrication processes will be critical for overcoming the challenges of both methods in industrial applications.

### 2.3 Substrate wettability

The surface properties of substrates, whether hydrophilic or hydrophobic, significantly influence the crystal morphology, grain size, and film uniformity of perovskite thin films. Substrate surface energy and wettability not only determine the spreading and diffusion behavior of precursor solutions but also directly affect the distribution, rate, and quality of nucleation sites. As indicated by eqn (2), the surface energy (*γ*) of the substrate influences the nucleation energy barrier (Δ*G*_v_), where higher surface energy generally lowers Δ*G*_v_, facilitating nucleation. Therefore, tuning substrate surface properties is an effective strategy to regulate the nucleation process of perovskite thin films.

Hydrophilic substrates exhibit strong interactions with precursor solutions, enhancing the spreading of solutions on the substrate and promoting the uniform dispersion of precursor molecules, which facilitates nucleation and typically leads to a high nucleation density.^22,50^ High surface energy also accelerates ion diffusion and lowers the nucleation barrier, increasing the nucleation rate.^51^ However, excessive nucleation points can result in random grain orientations or even polycrystalline films, thereby reducing crystal uniformity and single-crystal quality.^52^ Simultaneously, precursor ions may be captured by the hydrophilic surface, slowing ion diffusion and crystal growth rates.^10,53,54^ In specific cases, the introduction of self-assembled molecular layer modifications (*e.g.*, MeO-2PACz in Fig. 2b)^55^ to hydrophilic substrates can maintain their hydrophilic properties, enhance interface adhesion, stabilize crystal structures, and promote the growth of large-grain single crystals.

In contrast, hydrophobic substrates exhibit poorer wettability, which suppresses excessive nucleation and facilitates lateral crystal growth along the substrate surface.^52,56^ Lower surface energy limits the over-expansion of solutions, resulting in reduced nucleation density.^22^ This is consistent with the concept from eqn (4), where a higher nucleation rate (*J*_0_) is influenced by stronger interactions between the substrate and the precursor molecules, resulting in a larger number of stable nuclei. During single-crystal growth, as precursor concentration decreases and the solution around nuclei becomes unsaturated, ions must diffuse rapidly to replenish the saturated solution and sustain crystal growth.^57^ On hydrophobic substrates like PTAA or PTFE, fewer nucleation points and faster ion diffusion contribute to improved uniformity and larger crystal sizes, enhancing film quality.^36,57^ Additionally, the lower friction between the hydrophobic surface and precursor solutions further promotes the crystal growth rate, as depicted in Fig. 2c.^58–60^ By applying hydrophobic surface modifications to substrates, nucleation distribution can be reduced, nucleation density controlled, and larger lateral crystals encouraged.^61^ Furthermore, functional layers such as self-assembled monolayers or P3HT^58^ can effectively tune surface energy and improve nucleation quality.

While hydrophilic substrates enhance nucleation rate, they may lead to smaller or randomly oriented grains. Hydrophobic substrates, on the other hand, help control nucleation density and promote the formation of larger grains and lateral crystal growth. Choosing suitable substrate properties requires balancing factors such as film size, nucleation rate, and application scenarios. This balance is captured by the concept of critical nucleus radius (*r*_c_) from eqn (3). By adjusting surface energy and controlling nucleation density, one can influence the *r*_c_, allowing for better regulation of crystal size and grain boundaries. For instance, hydrophobic substrates may be advantageous for large-area single-crystal films, while mildly hydrophilic or functionally modified hydrophilic substrates may be more effective for rapid nucleation or localized directional growth. By tuning hydrophilic and hydrophobic characteristics and applying selective chemical modifications, it is possible to achieve high-quality, low-defect perovskite SCTFs suitable for large-area fabrication.

### 2.4 Component strategies

The appropriate ionic doping can significantly influence the nucleation barrier, the distribution of active sites, and the structural stability of perovskite single-crystal films during the nucleation stage. Chlorine doping, for instance, has been shown to enhance nucleation uniformity by increasing the ionic mobility and stabilizing the chemical coordination of Cl^−^ at iodide sites. This interaction reduces random nucleation and promotes more ordered crystal orientation.^62,63^ The underlying mechanism involves the reduction of nucleation barriers due to the electronic structure of the Cl^−^ ion, which influences the local charge distribution at the nucleation site, making it easier for perovskite precursor ions to aggregate into a crystal structure. The higher ionic mobility of Cl^−^ allows for faster rearrangement of ions at the nucleation site, which in turn leads to more uniform nucleation events.

The small ionic radius and high stability of Cs^+^ make it an effective replacement for methylammonium (MA^+^) cations.^64^ In terms of nucleation, Cs^+^ affects the lattice strain and electrostatic potential within the perovskite crystal lattice. This modification leads to a lowering of the nucleation barrier by alleviating strain in the crystal structure. This reduction in lattice distortion creates a more favorable environment for stable nucleation to occur, which is crucial for large-scale single-crystal growth. Cs^+^ also helps stabilize the nucleation sites, which ensures a higher density of stable nucleation points, leading to the growth of uniform and defect-free crystals.

Building on the role of Cs^+^ and Cl^−^ doping, other ions offer additional benefits for regulating the nucleation process. In contrast, Pb^2+^ to Sn^2+^ substitution not only reduces the toxicity of the material but also introduces significant changes in the electronic environment around the nucleation sites. Sn^2+^ has a lower charge density compared to Pb^2+^, which leads to a modification of the electrostatic field around the nucleation site. This alteration lowers the nucleation barrier by facilitating the formation of more stable nuclei.^65,66^ However, Sn^2+^ also introduces additional defects, such as vacancies, which must be controlled carefully to avoid disrupting the uniformity of nucleation. This trade-off between stability and defect formation makes the use of Sn^2+^ as a dopant more complex, requiring precise control of doping concentrations to achieve an optimal balance between stable nucleation and defect minimization.

Similarly, Bi^3+^ doping stabilizes crystal structures by adjusting the nucleation barrier through its interaction with the perovskite lattice. The electronic configuration of Bi^3+^, similar to that of Pb^2+^, allows it to stabilize the nucleation sites and centralize nucleation events. This results in fewer but more stable nucleation sites, which promote the growth of larger, more uniform crystals. The reduction in the nucleation barrier due to Bi^3+^ doping helps to control the density of nucleation sites, leading to a more controlled and predictable crystallization process.^67–72^

The impact of dopants on nucleation mechanisms is not limited to ionic size or electronic configuration alone. Functional dopants, such as Mn^2+^ and Sb^3+^, have been explored for their ability to regulate nucleation while introducing magnetic and spin characteristics. Mn^2+^ doping has been shown to suppress carrier recombination pathways, which indirectly influences nucleation by affecting the charge transfer dynamics in the perovskite crystal. Similarly, Sb^3+^ doping optimizes the bandgap and energy-level alignment, which indirectly impacts the nucleation process by stabilizing the charge distribution in the crystal.^73–75^ These functional doping strategies not only expand the application scope of perovskite materials but also enable precise control of nucleation sites, facilitating doping optimization in complex systems. The adjustment of nucleation barriers through the careful selection and incorporation of dopants can help optimize the crystallization process, ensuring the growth of high-quality, large-area single crystals with minimal defects.

Beyond doping strategies, additive approaches such as polymer additives, have also emerged as important tools for regulating the nucleation process. By controlling the nucleation dynamics by influencing the viscosity, solubility, and surface tension of precursor solutions, polymer additives lead to more uniform nucleation and the formation of high-quality single crystals. These additives act synergistically with dopants to fine-tune the nucleation barrier and promote better control over the crystallization process, offering a complementary strategy to doping techniques.

Recent studies have demonstrated the effectiveness of polymer-assisted strategies in addressing both surface trap states and phase instability in perovskite single crystals. Polymer ligands such as PEG, PPG, PVA, and PAA have been shown to significantly enhance the stability of organic–inorganic hybrid perovskite (OIHP) growth solutions by coordinating with Pb^2+^ ions. This coordination stabilizes the solution under high supersaturation conditions and reduces nucleation rates, enabling high-quality, large-size crystal growth. The polymer's oxygen groups facilitate interactions with Pb^2+^, reducing impurity nucleation and enabling faster, controlled growth of OIHPs.^76^

Poly (methyl methacrylate) (PMMA) has been used to passivate surface defects and inhibit phase segregation in FAMACs perovskites. The Lewis basic units in PMMA effectively passivate surface traps, while the hydrophobic units prevent moisture ingress, thus stabilizing the crystal structure and preventing phase transitions typically observed in FA-Cs perovskite systems.^77^ Moreover, polystyrene (PS) has been shown to significantly influence the nucleation of MAPbI_3_ perovskite crystals. The interaction between PS and lead iodide (PbI_2_) in precursor solutions facilitates the formation of a cross-linked polymer-perovskite network, which promotes nucleation and slows down crystal growth, leading to improved crystal quality.^78^ PS also provides a thin hydrophobic layer on the surface of perovskite crystals, protecting them from environmental degradation factors such as moisture, air, and light.

The combined effects of dopants and additives play a crucial role in controlling the nucleation and growth dynamics of perovskite single crystals. Dopants influence the nucleation barrier through various mechanisms, including electrostatic modifications, lattice strain relief, and electron density adjustments, to create a more stable environment for nucleation, improving the quality and uniformity of the resulting crystals. Additionally, polymer additives offer complementary approaches by stabilizing crystal surfaces, preventing phase instability, and reducing defects. Together, these strategies enable precise control over the crystallization process, ensuring the growth of high-quality, defect-free perovskite single crystals suitable for large-scale applications.

## 3. Growth dynamics

Perovskite SCTFs are strongly influenced by the ionic characteristics and solution-processing properties of the material. After nucleation, the crystal growth rate (*R*_T_) becomes a critical factor in determining the film's quality and uniformity. The growth rate is closely related to the solution's supersaturation (Δ*C*), as described by the equation:

7 *R*_T_ = *k* × Δ*C*

Where *k* is the growth rate constant and Δ*C* represents the supersaturation. High supersaturation typically accelerates crystal growth by promoting nucleation, but excessive supersaturation accelerates disordered growth and defect formation, particularly in perovskites, where weak electrostatic interactions in the crystal structure increase susceptibility to point defects and distortions. The ionic nature of perovskites allows effective growth even at low supersaturation levels, distinguishing them from traditional covalent or metallic crystals.^79^

During isothermal growth, the growth rate is also influenced by the solution's concentration (*C*) and temperature (*T*). Their relationship is expressed as:

8 *C* = *k*′ × e^−Δ*H*/*RT*^

Where *k*′ is a constant, Δ*H* represents the enthalpy of dissolution, and *R* is the gas constant. For perovskites, elevated temperatures generally increase solubility, promoting a stable supersaturation state. However, their thermal sensitivity means excessive temperatures can lead to premature nucleation and degraded crystal quality. Thus, precise temperature control is critical to avoid thermal stability issues while ensuring uniform crystal expansion.

The choice and characteristics of solvents are especially crucial in perovskite single-crystal growth. The ionic nature of perovskite precursors makes solvent polarity and complexation properties pivotal. For instance, highly polar solvents like DMSO and DMF form stable complexes with lead halides, slowing down rapid crystal growth. This complexation behavior reduces local supersaturation, facilitating a more uniform crystal expansion in the early stages of growth.

### 3.1 Chemical composition

The chemical composition of perovskite precursors not only determines their compatibility with alternative cations but also significantly affects their crystallization dynamics and optoelectronic properties. Adjusting the proportion of different precursor components offers a powerful means to modulate crystal growth rates and material stability, enabling tailored fabrication of high-quality SCTFs for diverse applications.

A-site organic cations, such as FA^+^ and MA^+^, play crucial roles in these processes. FA^+^, with its high thermal stability and lower volatility, is preferable for applications requiring enhanced stability. Conversely, MA^+^, due to its higher volatility, is less suitable under harsh conditions but can accelerate nucleation through higher supersaturation during the initial growth stages.^36^ A strategic introduction of FA^+^ and MA^+^ in sequential stages—using a higher proportion of MA^+^ during nucleation and gradually transitioning to FA^+^ during crystal growth—can balance nucleation rates and crystallization stability. Advanced approaches, such as incorporating thermosensitive or photosensitive cations, may enable dynamic control of ion ratios in response to environmental stimuli, offering a novel optimization pathway yet to be fully explored in perovskite research.

Halide ions, such as iodide (I^−^) and bromide (Br^−^), significantly impact crystal growth rates and directionality. Partial Br^−^ incorporation has been shown to widen the bandgap,^80^ yet its role in single-crystal nucleation and ion redistribution remains underexplored. Gradual modulation of I^−^ and Br^−^ concentrations during different growth phases could establish compositional gradients, enabling bandgap tuning while mitigating transport limitations caused by uniform halide distributions.^81^ Furthermore, pseudohalides such as thiocyanate (SCN^−^) have emerged as promising substitutes for traditional halides like I^−^ and Br^−^. SCN^−^ demonstrates potential in enhancing solution processability, material stability, and bandgap engineering, thus expanding the functional landscape of perovskites.^82,83^

Optimizing the chemical composition of perovskite SCTFs involves achieving a dynamic balance between nucleation and growth, structural stability, and bandgap tailoring for specific optoelectronic functionalities. Mixed-cation and mixed-halide systems have shown remarkable improvements in perovskite film performance^84,85^ but face integration challenges, such as uneven crystallization and adverse stress responses. A combined approach of solution processing and vapor deposition, where one component dominates in the initial solution while another is introduced during vapor-phase growth, could refine compositional uniformity and growth directionality. Gradient compositional tuning, implemented in distinct nucleation and growth stages, may also reduce defect densities and enhance nucleation uniformity, paving the way for advanced single-crystal perovskite applications.

### 3.2 Solvent selection

The polarity of a solvent directly influences the solubility of precursors in perovskite synthesis, thereby affecting ion behavior and solution supersaturation. Polar solvents like DMF and DMSO dissolve perovskite precursors effectively, enabling uniform ion dispersion, which facilitates ordered nucleation and crystal growth.^81,86^ However, rapid evaporation of these solvents can result in uneven precursor distribution, affecting nucleation density and growth rate, leading to irregular or polycrystalline film surfaces.

In addressing the trade-offs, the best combination of solvent properties typically involves balancing the solubility and evaporation rate. Employing solvents that have a higher polarity and slower evaporation rates, such as a DMF–DMSO mixture, and optimizing their ratios can reduce evaporation rates while maintaining solubility, enabling stable crystal growth.^87^ In addition, incorporating high-boiling-point co-solvents, such as GBL (γ-butyrolactone)or NMP (*N*-methyl-2-pyrrolidone), can further slowdown the solvent evaporation rate, prolonging the precursor drying time and facilitating more orderly nucleation and growth.^88,89^ However, a lower evaporation rate may delay the crystallization process, which could lead to excessive solvent retention and potentially disrupt the crystal structure if not carefully managed. To mitigate this, solvents with slightly lower polarity, such as chlorobenzene or toluene, can be added to the mixture to regulate evaporation while still maintaining good solubility.^53,90^ Fine-tuning the solvent composition by mixing these solvents can strike a balance between ensuring effective precursor dissolution and preventing uneven evaporation. High-boiling-point solvents may also introduce residual solvent issues, affecting the final film's stability and morphology. Therefore, fine-tuning the solvent composition and optimizing processing conditions, such as spin-coating speed and post-annealing temperature, are necessary to mitigate these trade-offs. Furthermore, adjusting ambient conditions, such as temperature, humidity, and gas flow rate, can play a crucial role in solvent evaporation dynamics, allowing for additional control over the crystallization process.

Moreover, additives can play a crucial role in modifying solvent properties to optimize crystal growth. For example, introducing hydrogen-bonding additives, such as urea or thiourea, can slow down the evaporation rate by increasing solvent viscosity while also influencing the interaction between precursor species, leading to more uniform film formation.^91–93^ Similarly, polymer additives, such as polyethylene glycol (PEG) and polyvinylpyrrolidone (PVP), have been shown to modulate nucleation kinetics by controlling precursor–solvent interactions, thereby preventing the formation of inhomogeneous grains.^94–97^ Another effective strategy involves antisolvent-assisted crystallization, in which antisolvents such as chlorobenzene, toluene, or ethyl acetate are introduced to induce rapid nucleation while suppressing uncontrolled crystallization.^98,99^ This method is widely used to improve perovskite film uniformity and grain size, although careful tuning of the antisolvent addition timing is required to avoid excessive phase segregation or film defects. By carefully selecting and optimizing solvent systems, additive strategies, and environmental conditions, researchers can mitigate the trade-offs between solvent properties and achieve high-quality perovskite single crystals with minimal defects.

The choice of solvent also impacts ion diffusion during crystal growth. Solvent polarity and molecular structure dictate interactions with precursor ions, influencing diffusion rates and nucleation behavior. Solvents like PbAc_2_, ethanol, and isopropanol exhibit strong coordination with perovskite precursor ions (Pb^2+^, I^−^), altering solubility and ion migration dynamics.^27,100–102^ Selecting solvents with moderate interaction strength can prevent undesirable reactions, ensuring controlled nucleation and growth.

Adjusting solubility and growth rates is essential for high-quality thin films. Adding antisolvents, such as hexane or isopropanol, can rapidly reduce solubility, inducing orderly perovskite crystal growth. This strategy minimizes nucleation rates, prevents premature crystallization, and allows slow crystal growth, reducing polycrystalline formation.^103^

Controlling the precursor solution's volume and delivery method further affects film quality. Excess solution can lead to polycrystalline growth, while insufficient solution coverage results in uneven films.^58^ Techniques like capillary flow deposition utilize capillary forces to regulate solution flow and distribution, minimizing waste and ensuring uniform film growth.^59^ Slow flow rates improve solution stability, reducing uneven nucleation and enhancing crystal quality.

### 3.3 Temperature control

The control of temperature during perovskite single-crystal thin film growth plays a critical role in balancing nucleation and crystal growth rates. Excessively rapid heating or high temperatures can accelerate nucleation, leading to the formation of numerous small grains and subsequently reducing the overall crystal quality. Conversely, overly low temperatures can minimize defects but slow the growth process, making it challenging to achieve large-area films.^104,105^ Gradual temperature increase strategies provide a balanced approach, enabling the simultaneous formation of high-quality and large-area crystals while minimizing the generation of small grains.

Gradual heating allows sufficient time for monomers in the precursor solution to achieve thermal equilibrium, promoting a more uniform crystal distribution and minimizing particulate defects. This approach facilitates the growth of high-quality, large-area perovskite crystals.^25,58^ Additionally, stable heating with lower rates minimizes temperature-induced fluctuations that often lead to defects or polycrystalline formations.

A moderate temperature reduction during the heating process can lower the supersaturation of the precursor solution, effectively dissolving smaller crystals and promoting the growth of larger, more complete crystals. In GHN-RTG technique, minor cooling can regulate the nucleation process, preventing excessive nucleation and avoiding the formation of grain boundaries or irregular crystals.^46^

Temperature gradients can further enhance the process by inducing convection between the substrate and the solution.^38^ This ensures a continuous supply of precursor materials, influences solution flow patterns, and aligns the crystal growth direction. Precise temperature distribution, particularly with multi-zone temperature setups, maintains lower solution supersaturation while ensuring ion transport and material supply during crystallization. However, refining temperature control to prevent local supersaturation or uneven heating remains critical for improving single-crystal thin film quality.

## 4. Film morphology and structure

In practical photovoltaic applications, the macroscopic properties of perovskite thin films—such as overall morphology, thickness control, and uniformity over large areas—directly impact device performance and require systematic optimization. Key to this problem is promoting lateral crystal growth, allowing the retention of single-crystal quality while adapting film thickness and orientation to meet the photovoltaic device's requirements for efficient charge-carrier pathways. With such a comprehensive approach, films can achieve superior optoelectronic performance, laying the groundwork for large-scale deployment of perovskite photovoltaic technologies. This section explores how to regulate the macroscopic structure and morphology of SCTFs to meet the demands of photovoltaic devices, focusing on enhancing power conversion efficiency and long-term stability.

### 4.1 Crystal orientation

**Fig. 3:**
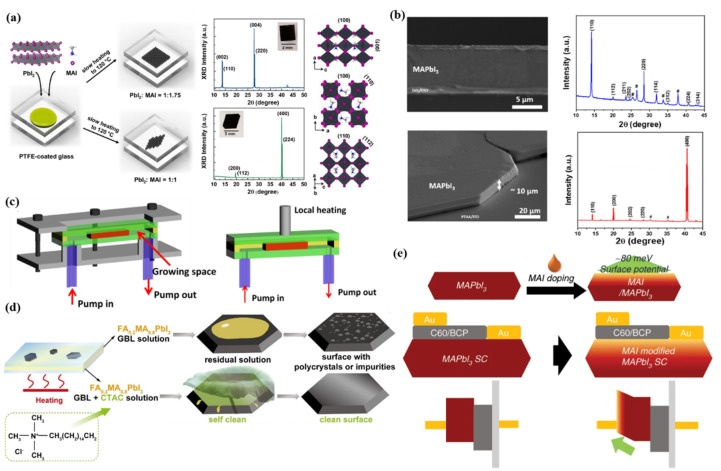
(a) Schematic of the fabrication of MAPbI_3_ SCTF between PTFE-coated glasses, showing preferred (001) and (100) orientations achieved by precursor ratio control. XRD patterns confirm orientations, with inset photographs of films and crystal structure views.^108^ (b) SEM surface and cross-sectional images and XRD patterns of MAPbI_3_ film grown on TiO_2_/FTO and PTAA/ITO respectively.^58^ (c) Schematic diagram of growth module and local heating with homothermal aluminum block for MAPbBr_3_ SCs.^21^ (d) Schematic illustration of FA_0.2_MA_0.8_PbI_3_ thin single crystals grown by the space-confined method with and without CTAC.^122^ (e) Schematic diagram of device structures and energy levels for SC-PSCs without and with MAI treatment.^124^

The crystallographic orientation of perovskite SCTFs has a direct impact on the migration pathways of electrons and holes. Therefore, during the growth process of perovskite SCTFs, adjusting growth conditions, especially through post-treatment methods such as thermal annealing or introducing interface layers, can effectively control the crystal orientation. Studies have shown that perovskite thin films with (100) or (110) orientations exhibit higher carrier mobility, facilitating efficient electron and hole transport, reducing carrier recombination, and ultimately improving the photovoltaic conversion efficiency of devices.^106,107^ Controlling crystal orientation to grow films along preferred directions such as (100) or (110) has become an important strategy to enhance the efficiency of perovskite photovoltaic devices. Yang *et al.* synthesized perovskite SCTFs using a space-constrained method, successfully obtaining films with different orientations by adjusting the precursor concentration (Fig. 3a).^108^

During the growth of perovskite thin films, charge transport layers (*e.g.*, TiO_2_, PTAA) significantly influence crystal orientation, and introducing interface layers is an effective way to optimize orientation. For instance, MAPbI_3_ films grown on TiO_2_ exhibit a polycrystalline orientation along the (110) direction, whereas those grown on PTAA exhibit single-crystal orientation along the (400) plane, as depicted in Fig. 3b.^58^ These differences arise from the chemical and crystallographic compatibility between the charge transport layer and the perovskite material, which subsequently affects the growth direction of the film. Selecting appropriate charge transport layers and precisely tuning their structures can effectively guide perovskite films to grow in preferred directions, reducing disordered grain boundaries and promoting efficient charge transport.

Despite significant progress in optimizing the crystal orientation of perovskite SCTFs, many unresolved questions remain about its profound impact on overall film performance. In particular, achieving ideal orientation with consistent film quality during large-scale production remains challenging. Exploring multi-level synergistic regulation strategies, such as combining thermal annealing with interface layer design, could optimize orientation during both growth and post-treatment stages. Beyond traditional interface materials like TiO_2_ and PTAA, zinc tin oxide (Zn_2_SnO_4_) and its composites (*e.g.*, Zn_2_SnO_4_–SnO_2_) have shown potential for optimizing perovskite orientation.^109–111^ These materials offer excellent band alignment and crystallographic compatibility, reducing interfacial defects and promoting crystal growth in specific directions. Additionally, hybrid structures combining perovskite nanocrystals and 2D materials are considered promising options, as they guide crystal growth along preferred orientations, improve film quality, and enhance photovoltaic performance.^112^ Furthermore, building on interface materials and post-treatment methods, combining various optimization approaches could simultaneously enhance optoelectronic performance and film stability, laying a stronger foundation for the large-scale application of future photovoltaic devices.

### 4.2 Thickness control

To grow large-area, thin perovskite single-crystal films, researchers focus on regulating growth parameters such as temperature, solution concentration, composition, and spatial design while enhancing optoelectronic performance and device stability. A promising approach involves confined space growth techniques, such as sandwich structures or micron-scale gaps. These methods limit solution diffusion in the vertical direction and promote lateral crystal growth. For instance, a growth space using polytetrafluoroethylene (PTFE) boundary frames can control film thickness by adjusting the PTFE layer thickness, as depicted in Fig. 3c.^21^ However, vertical geometrical constraints often reduce precursor solution transport efficiency, restricting the continuous supply of solute to nucleation points and limiting lateral crystal size.^113^ To address this, hydrophobic spacers or temperature gradient application can optimize the confined growth environment.^47^

For film thickness control, the capillary-driven immersion growth method has shown significant advantages. Two clean substrate plates are sandwiched and immersed in precursor solutions, where capillary action ensures even solution distribution. Thickness can be finely tuned based on substrate spacing to nanoscale or microscale levels.^38^ Furthermore, growth inhibitors like oleic acid (OA) effectively suppress vertical growth, promoting enhanced lateral expansion.^114^

Although thinner films reduce carrier migration pathways and improve photovoltaic efficiency, they exacerbate surface recombination effects. To mitigate this, optimizing solution concentration, substrate surface properties, and temperature gradients during fabrication is crucial. Such optimizations reduce defect density while maintaining high film uniformity and excellent optoelectronic performance. Incorporating differential space-limited crystallization (DSLC) techniques with hydrophobic structures in the growth space can regulate solvent evaporation rates and ensure uniform lateral crystal growth.^57^

### 4.3 Surface treatment

During the growth process of perovskite SCTFs, residual solution or solvent often affects the surface uniformity and stability of the film, which in turn negatively impacts the performance of photovoltaic devices. Particularly in solution-based growth methods, residual precursor solution or solvent can cause surface defects, increase the likelihood of carrier recombination, and reduce the electronic transport efficiency and stability of the device.^27,115,116^ Therefore, effectively removing these residues and applying surface treatments can not only improve the surface quality of the films but also passivate surface defects, optimize the interface characteristics between the film and the electrode, and ultimately enhance the photoelectric performance and long-term stability of the films.

To address this issue, surface engineering techniques such as surface cleaning, plasma treatment, and slow drying have become common approaches for removing residual solutions.^117^ Plasma treatment can effectively eliminate solvent molecules on the surface while improving the surface structure of the film. This can enhance its hydrophilicity or hydrophobicity, thereby promoting uniform crystal growth and improving surface quality. Additionally, slow drying helps remove excess solution from the crystal surface and prevents the formation of unnecessary polycrystals or impurities, maintaining the integrity of the perovskite crystal.

Passivation treatment methods that introduce long-chain organic molecules or oxide coatings can effectively passivate surface defects and interfaces, significantly improving the photoelectric performance of perovskite films.^118–121^ Due to the ionic nature of perovskite materials, the crystal surface often exhibits certain hydrophilicity, which can lead to residual solution on the surface and the formation of polycrystals. Amphiphilic compounds such as CTAC (cetyltrimethylammonium chloride) utilize their hydrophilic groups to interact electrostatically with the perovskite surface, while the hydrophobic groups remain on the exterior. This structure enhances the hydrophobicity of the single-crystal film surface, facilitating the spontaneous removal of growth solution and reducing the formation of polycrystals, as depicted in Fig. 3d.^122^

In the surface treatment of perovskite SCTFs, beyond the aforementioned chemical processes, further enhancement of photovoltaic device performance can be achieved by adjusting the interfacial characteristics between the film and the electrode. In perovskite solar cells, the contact between the anode and the surface of the perovskite thin film often exhibits a significant work function mismatch, which can result in interfacial energy level misalignment and hinder effective electron transport.^123^ To address this issue, a simple surface treatment involves spin-coating a diluted MAI solution on the film to modify the ratio of MAI to PbI_2_, as depicted in Fig. 3e.^124^ This approach optimizes surface conductivity and aligns interfacial energy levels. Moreover, this straightforward process not only improves the contact between the film and the electrode but also repairs surface damage caused by residual solution. It passivates the surface defects associated with methylammonium cations, enhances carrier transport, suppresses non-radiative recombination, and further improves the film's stability and photoelectric performance.

## 5. Conclusions and perspective

Perovskite SCTFs, with their high crystallinity and low defect density, exhibit immense potential in photoelectric performance, making them an ideal material for photovoltaic devices. The relatively simple fabrication process and low production costs further endow them with significant commercial advantages for future photovoltaic applications. This review systematically analyzes the impact of nucleation control, growth dynamics, and post-processing techniques on SCTF quality and performance. It aims to provide theoretical guidance for manufacturing SCTFs in photovoltaic applications. By exploring the influence of key factors on thin-film quality at a mechanistic level, the review seeks to optimize the performance and stability of perovskite photovoltaic devices, advancing their broad application and commercialization.

Achieving uniform nucleation is fundamental to growing high-quality SCTFs. By precisely tuning solution saturation, substrate surface treatment, and chemical composition, the density and distribution of nucleation sites can be effectively controlled, promoting the formation of large-grained, low-defect single-crystal structures. The introduction of real-time monitoring technologies can provide deeper insights into *in situ* nucleation behavior, enabling dynamic adjustments during the nucleation stage for more flexible control of nucleation rates and crystal orientation.

Factors such as temperature, solvent selection, and substrate properties play critical roles in the growth dynamics of SCTFs. Optimizing the combination of these parameters facilitates the production of uniform, dense perovskite SCTFs while minimizing defect density. However, striking a precise balance among these factors remains a significant challenge. To address this, further development of growth techniques that integrate temperature gradients and solvent evaporation rates is recommended, aiming for large-area uniform thin-film growth.

Enhancing the crystal orientation and passivating surface defects of SCTFs often relies on post-processing techniques such as annealing and interface engineering. Introducing passivation layers and selective interface passivation techniques not only reduces defect density in the thin film but also improves carrier transport efficiency, thereby boosting the performance and environmental resilience of photovoltaic devices. Despite these advancements in material performance, the commercial application of SCTFs still faces several challenges. While techniques such as confined-space crystallization and microchannel-assisted growth have demonstrated potential for large-area thin-film production, further optimization is required to improve efficiency in scaled-up manufacturing processes. Moreover, stability issues under long-term operational conditions—such as humidity, temperature fluctuations, and UV exposure—remain critical problems to be addressed. Future research should focus on developing durable encapsulation techniques, stable interfacial materials, and chemically robust inorganic/organic hybrid perovskite materials to enhance the aging resistance of SCTFs and accelerate their widespread adoption in practical photovoltaic applications. In particular, scaling up methods such as space-confined growth, which could enable controlled crystallization in large-area panels, and inorganic passivation strategies, which improve stability, offer significant potential for commercialization and large-scale deployment.

Perovskite SCTFs represent a promising frontier in photovoltaic and optoelectronic research, combining the structural advantages of single crystals with the scalability of thin-film technologies. This review has highlighted the intricate interplay of nucleation dynamics, growth kinetics, and post-growth optimization in determining SCTF quality. Key insights include the role of precise nucleation control in reducing defects, the impact of growth dynamics on achieving uniform and large-area films, and the critical importance of post-growth processing in enhancing stability and optoelectronic performance. To further improve the commercialization potential of SCTFs, future research must prioritize scalable growth techniques and explore methods such as space-confined growth and inorganic passivation for large-area, high-performance panels.

The full potential of SCTFs requires addressing several interconnected challenges. These include refining growth methodologies to ensure consistency across large areas, engineering robust compositions and doping strategies for enhanced performance, and developing reliable encapsulation and interfacial solutions to mitigate environmental degradation. Moreover, leveraging advanced *in situ* monitoring techniques could provide a deeper mechanistic understanding and real-time optimization of the growth process. By integrating these approaches, further research can not only overcome the current limitations but also expand the applicability of SCTFs in practical devices.

## Data availability

No primary research results, software, or code have been included and no new data were generated or analysed as part of this review.

## Author contributions

Conceptualization, R. W., and J. X.; writing – original draft, J. S.; writing – review & editing, R. L., Y. G., X. S. and R. W.; supervision, R. W. and J. X.

## Conflicts of interest

The authors declare no competing interests.
